# A Positive Psychology Perspective on Positive Emotion and Foreign Language Enjoyment Among Chinese as a Second Language Learners Attending Virtual Online Classes in the Emergency Remote Teaching Context Amid the COVID-19 Pandemic

**DOI:** 10.3389/fpsyg.2021.798650

**Published:** 2022-01-12

**Authors:** Qing Wang, Yuhong Jiang

**Affiliations:** Foreign Languages College, Shanghai Normal University, Shanghai, China

**Keywords:** positive psychology, foreign language enjoyment, Chinese as a second language, language students, online classes, emergency remote teaching, COVID-19

## Abstract

This study adopted a positive psychology perspective to investigate positive emotion and foreign language enjoyment among Chinese as a second language (CSL) learners in an emergency remote teaching (ERT) context amid the COVID-19 pandemic. A set of 90 preparatory Chinese language students (40 males and 50 females) was assessed for their level of foreign language enjoyment using the Foreign Language Enjoyment Scale (FLES). Participles' scores on self-perceived language achievement and actual test scores were adopted as the measurement of their Chinese language proficiency. The results revealed that: (1) CSL learners experienced high level of FL enjoyment in an online learning context, (2) no significant correlation was found between FLE and leaners' actual language achievement nor between FLE and their self-perceived achievement, (3) female learners showed higher FLE than male learners and gender was found to have a significant effect on FLE-Private, (4) participants' geological location, i.e., whether in China or at home countries, significantly influenced their FLE, (5) participants' regional group was not a significant predictor of FLE, and (6) teacher-related variables and learner self-perceptions of achievement were strong predictors of FLE among CSL learners. The findings highlight the importance of teacher's role in an online learning environment and suggest that FLE may not boost performance in the short term for language beginners but is still conducive in the long run. Implications for both teachers and learners, and suggestions for future researches are provided.

## Introduction

Positive psychology (PP) is the empirical study of how people thrive and flourish, with the aim to help people lead better lives and catalyze building positive qualities in life rather than focus solely on repairing the worst things (Seligman and Csikszentmihalyi, 2000; MacIntyre and Mercer, 2014). The advent of modern positive psychology can be traced back to the presidential address to the *American Psychological Association* in 1998 given by Martin Seligman who, as a leading advocate of PP, used his APA presidency to initiate a shift in psychology's focus toward a more positive psychology (Seligman, 1999; Linley et al., 2006). Positive psychology contributes another perspective to psychology by studying what we can do to increase strengths and attributes such as resiliency, happiness, optimism and the like in the general population. And the workings of positive internal experiences such as emotions are one of the topic areas positive psychology has been designed to address since its inception (MacIntyre and Mercer, 2014).

Prior studies on emotions in SLA and language learning have developed a fixation upon investigating negative emotions such as anxiety, which has been the most widely studied emotion (MacIntyre and Gregersen, 2012; Dewaele and MacIntyre, 2014), and has proved to be negatively related to second or foreign language achievement (Horwitz, 1986; Horwitz et al., 1986; Aida, 1994; Ganschow and Sparks, 1996; Cheng et al., 1999). Suggestions and recommendations have been springing concerning anxiety reduction, and the reducing of negative and unpleasant feelings to alleviate their disruptive influence, given the almost consistent negative correlation between anxiety and language learning (Young, 1991; Dewaele and MacIntyre, 2014; Gregersen and MacIntyre, 2014).

Influenced by the positive psychology movement, many researchers, in recent years, have embarked on the journey to include positive emotion, enjoyment in this regard, in their researches on second or foreign language learning (Dewaele and MacIntyre, 2014, 2016, 2019; Dewaele et al., 2016, 2017; Dewaele and Dewaele, 2017; Boudreau et al., 2018; Jiang and Dewaele, 2019; Zhang and Tsung, 2021). MacIntyre and Gregersen (2012) argued that positive emotions function differently from negative emotions with the former facilitating while the latter debilitating language learning. Later on, MacIntyre and Mercer (2014) acknowledged that positive psychology (PP) helps broaden and enrich research themes in SLA and thus called for the adoption of a PP perspective which helps SLA researches to take a holistic view of both positive and negative emotions, highlighting second language learners' non-target-like performance and the whole of their L2 performance and knowledge instead of fixing on their learning deficits (Dewaele and Dewaele, 2017; Dewaele et al., 2017). In light of the situation, Dewaele and MacIntyre (2014) examined a positive emotion (enjoyment) and a negative emotion (anxiety) in foreign language learning and investigated their relationship. Their results revealed that foreign language enjoyment (FLE), a common positive emotion experienced by L2 learners (Piniel and Albert, 2018), is positively associated with L2 achievement, and led to the conclusion that FLE and foreign language anxiety (FLA) are different dimensions and not two sides of the same coin (Dewaele and MacIntyre, 2014).

Later emotion researches in second or foreign language learning, on the one hand, have grown an appetite for fostering positive emotions and reducing negative ones to facilitate language learning, uncovering the effect of multifaceted variables on positive and negative emotions as well as their relationship with various target learners (Dewaele and MacIntyre, 2016; Dewaele et al., 2017, 2019; Dewaele and Dewaele, 2018; Jiang and Dewaele, 2019; Moskowitz and Dewaele, 2020). On the other hand, the perspectives of emotion researches in language learning are being further explored and the research methodologies are becoming all the more innovative. Elahi Shirvan and Taherian (2020), for instance, explored the actualization of potential affordances for FLE. They adopted a modern social hermeneutics approach and, from an ecological perspective, came up with accurate interpretations regarding the contextual process of the actualization of affordances for FLE within the microsystem of the classroom. In the meantime, the spectrum of dynamic perspective also embraces varied approaches into emotion researches in language learning, such as the idiodynamic approach (Elahi Shirvan and Talebzadeh, 2018; Elahi Shirvan and Talezadeh, 2018), the use of self-organizing maps (De Ruiter et al., 2019), the use of retrodictive qualitative modeling (Elahi Shirvan and Talebzadeh, 2020), and the use of ecological momentary assessment (Elahi Shirvan et al., 2020). Moreover, studying from a longitudinal perspective and carrying out longitudinal designs, which are better at establishing causality (Dewaele and Li, 2020), also yields profound research significance and implications in regard to emotion and language learning (e.g., Elahi Shirvan and Taherian, 2021; Elahi Shirvan et al., 2021).

Nevertheless, the majority of researches on emotions, both positive and negative, in second or foreign language learning has targeted learners of Indo-European languages, EFL in particular. Given the surging trend of learning Chinese in this new millennium and the burgeoning number of researches on learning and teaching Chinese as a second or foreign language, however, Zhang and Tsung (2021) called for the urgent need to study emotions experienced by Chinese as a second language (CSL) learners in their language learning process and their study is probably one of the first to examine FLE among CSL learners. Meanwhile, few studies have previously involved language learners in an emergency remote teaching (ERT) context, which is an alternate delivery of teaching during crisis circumstances, manifested by a sudden shift of teaching venues from “in-person” to online in the wake of the global COVID-19 pandemic (Resnik and Dewaele, 2021).

The present study adopts a positive psychology perspective to investigate positive emotion and foreign language enjoyment among Chinese as a second language (CSL) learners who were learning the language in an emergency remote teaching (ERT) context due to the current global COVID-19 pandemic that has denied their physical access to attend offline face-to-face and “in-person” classes in China. We hope the findings of this study will enrich relevant FLE studies concerning CSL learners and offer important implications for the teaching and learning of Chinese as a second or foreign language in China and in the global context, particularly in an ERT context.

## Literature Review

### Emotions and Language Learning

Conceptualizing emotions as “multicomponent response tendencies that unfold over relatively short time spans,” Fredrickson (2001) made a proper differentiation in the function between positive and negative emotions. Later, Reeve's (2005) offered a multidimensional definition of emotions as “short-lived, feeling-arousal-purposive-expressive phenomena that help us adapt to the opportunities and challenges we face during important life events” (p. 294). MacIntyre and Gregersen (2012) took Reeve (2005) definition of emotions and viewed it from four components: subjective, arousal, purposive and expressive. They contended that emotions must be “understood to be more than the sum of these parts,” and that an emotion is “emergent from the coordination of these four aspects of experience” (p. 195).

Emotion, as has been unanimously acknowledged, is an affective reaction that changes the way of thinking, behaving and expressing (Scherer et al., 2001; Pishghadam et al., 2016b). In the same vein, emotion also exerts significant impacts on education and learning (Pishghadam et al., 2016b). Pekrun et al. (2002a) investigated emotions in an educational setting and defined these emotions as achievement emotions (Pekrun, 2006), which have strong influence on learners' motivation, learning strategies, identity development, and health (Schutz and Pekrun, 2007; Pishghadam et al., 2016b). In second or foreign language learning, learners also experience certain amounts of emotions which are considered to play a key role in the learning process (Zhang and Tsung, 2021). Pishghadam and Zabihi (2012) contended that “emotional ability is one of the indicators of improving the quality of life and, therefore, teaching should not focus merely on a specific subject or domain but should also include emotions” (Pishghadam et al., 2016b, p. 512). In this regard, Pishghadam et al. (2013), referring to Greenspan's (1992) Developmental Individual-Difference Relationship-Based model (DIR), set forth a new approach to SLA, Emotion-Based Language Instruction (EBLI), which “is based on the fact that having stronger emotions toward second/foreign language vocabularies leads to a better understanding of them and facilitates learning” (Pishghadam et al., 2016b, p. 513). They also introduced the concept of emotioncy, which refers to the degree of emotions one has toward language entities (Pishghadam et al., 2013), and is defined as “the varying degrees of sensory emotions that each entity (either a word or a concept) evokes and carries for each individual, depending on whether they have heard about, seen, touched or experienced that entity in their own context” (Pishghadam et al., 2019, p. 44). Emotioncy “ranges on a hierarchical order of null, auditory, visual, kinaesthetic, inner, and arch emotioncies” (Pishghadam, 2015, p. 1), and “higher levels of emotioncy (inner and arch) bring about higher levels of comprehension, learning, and retention because of involvement, i.e., they engage learners from inside, while lower levels of emotioncy (auditory, visual, kinaesthetic) lead to exvolvement because they engage learners from outside” (Pishghadam, 2015; Pishghadam et al., 2016b, p. 513). Bearing the EBLI and the concept of emotioncy in mind, Pishghadam et al. (2016b) set out to examine EFL emotions in English language classes and the results of their study uncovered that English language skills, namely, listening, speaking, reading and writing, were connected to a series of emotions and that “it is essential to help students manage, regulate, and control their emotions and feelings in language classrooms” to facilitate language learning (p. 521).

Dewaele and Li (2020) divided emotion research in SLA into three broad phases according to the major types of emotions studied in each phase: Emotion Avoidance Phase, Anxiety-Prevailing Phase, and Positive and Negative Emotions Phase. In the first phase, roughly between 1960s and the mid 1980s, emotions were considered as irrational factors in language learning and therefore did not receive much attention (Prior, 2019). The second phase of SLA research in Dewaele and Li (2020), starting from the mid 1980s, overlaps with the humanistic movement in language teaching which was at its peak in the 1970s and 1980s (MacIntyre and Mercer, 2014). The humanistic language teaching stresses the need to unite the cognitive and affective domains in order to educate the whole person, dealing with learners and their particular cognitive and affective nature and needs (Arnold, 1999). In proposing his Affective Filter Hypothesis, Krashen (1985) also drew attention to the role of emotions. He argued that an affective filter would be raised when the learner is experiencing a high degree of negative emotion such as anxiety, preventing the amount of comprehensive input from entering his language acquisition device (LAD) and thus debilitating language learning. However, the filter would be weaker or lower when the learner is more positive or experiencing positive emotions, which allows more input to be obtained and facilitates better language performance. As stated by Dewaele and Li (2020) in the second phase of SLA research, investigation on the negative emotions and their debilitative effects have gained strong ground, largely owing to the fact that negative emotions often reflect immediate problems or objective danger, forcing people to take actions accordingly, and therefore are more urgent and may override positive emotions (Seligman and Csikszentmihalyi, 2000). As a result, much territory of studies on positive emotions has been left uncharted and neglected. However, upon entering the third phase of Positive and Negative Emotions Phase in the 2010s, researchers began to develop interest in the psychology of learning (Dewaele and Li, 2020). MacIntyre and Gregersen (2012), based on the broaden-and-build theory of positive emotions developed by Barbara Fredrickson that underscores the ways in which positive emotions are essential elements of optimal functioning (Fredrickson, 1998, 2001, 2003, 2004), drew the attention to positive and negative emotions in a language classroom setting and conceptualized emotion along two separate dimensions: positive-broadening and negative-narrowing. MacIntyre and Gregersen acknowledged that negative emotions in SLA, anxiety in particular, had already been well-documented in the literature and lamented the lack of relevant researches on the powerful effects of positive emotions. Positive emotions, according to Fredrickson (2004), can “broaden an individual's momentary thought-action repertoire and build that individual's personal resources; ranging from physical and intellectual resources, to social and psychological resources” (p. 1367). Motivated by the “positive renaissance” led by positive psychology and the strengths of positive emotions (MacIntyre and Gregersen, 2012), MacIntyre and Mercer (2014) introduced the concept of positive psychology (PP) into SLA researches and believed that PP “opens up a broad, rich collection of under-researched themes” in SLA field, which is “perhaps in a particularly strong position to engage with PP to generate innovative thinking and research” (p. 167).

### The Development of Foreign Language Enjoyment and Its Measurement

Enjoyment, in positive psychology, refers to “the good feelings people experience when they break through the limits of homeostasis—when they do something that stretches them beyond what they were—in an athletic event, an artistic performance, a good deed, a stimulating conversation” and it is a state of mind that leads to personal growth and long-term happiness (Seligman and Csikszentmihalyi, 2000, p. 12). Enjoyment is the emotion felt when one not only meets their needs but also surpasses them to accomplish something unexpected or surprising (Csikszentmihalyi, 2008; Boudreau et al., 2018). In their control-value theory of achievement emotions, Pekrun et al. (2007) used a three-dimensional taxonomy and revealed that enjoyment, a major component of achievement emotions, is a positive, activating and activity-focused emotion that related positively to students' academic achievement (Pekrun et al., 2002b).

Foreign language learners experience a range of positive emotions in their learning process, such as enjoyment, hope, pride, etc. (Piniel and Albert, 2018). Of the positive emotions encountered, enjoyment has been found to be the most frequently experienced positive emotion, as opposed to anxiety (Dewaele and MacIntyre, 2014, 2019; Li, 2018; Pavelescu and Petrić, 2018; Piniel and Albert, 2018; Dewaele and Li, 2020). Dewaele and MacIntyre (2014) was the first to introduce foreign language enjoyment (FLE) to this field. They set off to examine both positive (foreign language enjoyment) and negative (foreign language anxiety) emotions, and collected quantitative and qualitative data from a large sample of foreign language learners (*N* = 1,746) who experienced higher levels of enjoyment than anxiety. Their research results showed that foreign language anxiety and foreign language enjoyment were negatively correlated (*r* = −0.34) but shared only 12.9% of their variance which was a small effect size, leading to their conclusion that FLE and FLCA are “independent emotions and not opposite ends of the same dimension,” and that “the absence of enjoyment does not automatically imply a high level of FLCA, and an absence of FLCA does not mean a presence of FLE” (Dewaele and MacIntyre, 2014, p. 261). In their large sample study, Dewaele and MacIntyre (2014) developed an instrument to measure foreign language enjoyment based upon Ryan et al. (1990) Interest/Enjoyment subscale. The original scale contained 21 items reflecting various facets of FLE and a positive environment in the FL class. In a series of subsequent and consecutive studies, Dewaele and MacIntyre (2016) shortened and reformulated the original scale and came up with a 14-item foreign language enjoyment scale. This version of scale focused on two FLE factors: FLE Social and FLE Private, with the former defined as the “positive feelings, encouraging peers, nice teachers and a supportive environment” and the latter as the “thoughts and feelings coalescing around a sense of accomplishment” (p. 225, 228). Later, with a UK sample of participants, Dewaele and Dewaele (2017) further extracted the FLE scale in Dewaele and MacIntyre (2014) to 10 items and added a third FLE dimension, namely peer-controlled vs. teacher-controlled positive atmosphere in the FL classroom.

Dewaele and MacIntyre (2014, 2016), Dewaele and Dewaele (2017), Dewaele and Alfawzan (2018), and Jiang and Dewaele (2019) have contended that foreign language enjoyment, as well as foreign language anxiety, is associated with a number of socio-biographical variables, such as participants' cultural background and disposition, teachers' professional and emotional skills, and students' attitude toward the teacher, etc. Bearing this in mind, Li et al. (2018), for instance, investigated foreign language enjoyment among Chinese high school learners of English. They confirmed and validated a 11-item and 3-factor (FLE-Private, FLE-Teacher, and FLE-Atmosphere) model of FLE as the measurement of research. Most recently, Botes et al. (2021) developed a short form of Foreign Language Enjoyment Scale (S-FLES) with learners of English, French and Spanish. They set forth a 9-item S-FLES with a 3-factor hierarchical model encompassing teacher appreciation, personal enjoyment, and social enjoyment. The shortened FLE scale, according to Botes et al. (2021), “provides a valid and reliable short-form measure of FLE, which can easily be included in any battery of assessments examining individual differences in FL learning” (p. 1).

The employment of the FLES in languages other than English (LOTE), Chinese as a second language in particular, has been peripheral and only a very limited number of researches has involved international learners of Chinese in a Chinese context (e.g., Chen et al., 2021; Zhang and Tsung, 2021). Nonetheless, as Li et al. (2018) perfectly demonstrated, more international samples concerning different social and cultural groups and different languages are urgently needed in a bid to further explore FLE, so that we can get a full picture of different learners' FL enjoyment and thereby boost their positive emotions to facilitate language learning.

### Learner Emotions and Foreign Language Enjoyment in Online Teaching Amid Crisis

The global COVID-19 pandemic starting from the early 2020 has caused a sudden shift of educational venues from “in-person” face-to-face teaching to online teaching due to the fact that mass gatherings have been prohibited and that quarantine and lockdowns have been imposed in an effort to contain the spread of the virus. Such phenomenon or new unique teaching context has been termed as “emergency remote teaching” (ERT) which is defined as “a temporary shift of instructional delivery to an alternate delivery mode due to crisis circumstances” (Hodges et al., 2020; Resnik and Dewaele, 2021). The definition of ERT distinguishes itself from planned online causes in terms of the time for implementation of educational activities. To investigate learner emotions, either positive or negative ones, amid the COVID-19 pandemic in an ERT context as opposed to regular offline face-to-face classes, and to unveil the possible underlying effects that the sudden shift of educational venues has exerted on learner emotions have been urgent since “ERT bears unique challenges for both teachers and learners alike” (Resnik and Dewaele, 2021). ERT is different from traditional “in-person” teaching in that it requires more from both the teacher and the learner. Teachers will have to devote more time, teaching skills and approaches in order to make the classes more effective, and learners will have to adapt and become more motivated, self-directed and goal-oriented and be less dependent on their teachers and more dependent on themselves (Kuong, 2011; Resnik and Dewaele, 2021). In addition to the added requirements from ERT, learners learning in an online setting may feel isolated which in turn can impose negative impact on their own satisfaction in learning experience in regular online classes (Kuong, 2015). The sense of being socially disconnected could exacerbate and become more salient in emergency remote teaching context amid the outbreak of a global pandemic when quarantines and lockdowns are imposed and social activities restricted (Resnik and Dewaele, 2021).

Resnik and Dewaele (2021) represents one of the first studies looking into learner emotions in both ERT and “in-person” face-to-face teaching contexts during an unprecedented global COVID-19 pandemic crisis in recent times. Their study examined foreign language enjoyment (FLE), as well as foreign language classroom anxiety (FLCA), among tertiary-level EFL students in Europe amid the COVID-19 pandemic that reduced learners to taking classes online. The results of their study were profoundly significant and yielded great implications in relation to the research of learner emotions in language learning amid pandemic and crisis periods. Their findings indicated that learners' emotions were less intense in ERT settings and that learners showed significantly lower level of FLE, as well as lower level of FLCA, in ERT than in “in-person” classes. They found out that “learners felt the ERT setting changed the classroom dynamics by making it more remote” and that “learners not being physically present in classrooms weakens all emotions, and breaks the relationship between them,” to which they attribute the possible explanation that “disembodied classes have less emotional resonance” (Resnik and Dewaele, 2021).

## The Present Study

This study was conducted in some local university in Shanghai, China, with the aim to investigate foreign language enjoyment (FLE) among international Chinese as a second language (CSL) learners whose Chinese courses were all carried out in an online context due to the global COVID-19 pandemic. The pandemic broke out at the beginning of the 2020s has greatly impacted, if not changed, the normal proceedings in which educational activities are carried out, with an increasing shift from offline face-to-face classes to online teaching and learning. The Ministry of Education in China issued a policy termed “Post-ponement of School without Suspension of Learning” (Chinese name: “停课不停学”) in early 2020, enabling schools to flexibly adopt online approaches to continue teaching and learning activities. The same solution was also applied to international students learning Chinese in Chinese universities during the time when China was going through critical period of the pandemic.

Although online teaching has almost, if not entirely, been lifted in mainland China owing to better control of the pandemic, international CSL students who remain in their home counties and therefore cannot come back to China for their studies have to continue their learning online. The same is true to those who are physically in China but whose fellow classmates are still at their home countries, since it is impractical to assign and teach the same class of students in two separate venues, i.e., online and offline.

### Participants

Participants in this study were 90 international CSL learners pursuing their studies in some local university in Shanghai, China. There were 40 males and 50 females, with an average age of 20.14 years (*SD* = 2.468) and an age range of 17–32. The majority of them came from Central, East and South Asia, Africa, with a few from West Asia and Europe. Participants registered in this study were all language students enrolled in an intensive Chinese language training program that aimed to prepare them linguistically for future studies in China. The majority of them have studied Chinese for less than a year and therefore can be regarded as almost beginners of Chinese. While having Chinese language as their compulsory course of study, they were also provided with other optional academic subjects such as math, chemistry, history, business Chinese, etc. Detailed socio-demographical information of our participants can be seen in Table 1.

**Table 1:** Socio-demographical information of the participants (*N* = 90).

**Variable**	**Category**	**Number**	**Percentage**
Gender	Male	40	44.4%
	Female	50	55.6%
Age group	17–19	44	48.9%
	20–22	35	38.9%
	23–25	7	7.8%
	26–32	4	4.4%
Regional group	West Asia	7	7.8%
	Central Asia	14	15.6%
	East Asia	24	26.7%
	South Asia	23	25.5%
	Africa	10	11.1%
	Europe	12	13.3%
Current location	In China	20	22.2%
	At home countries	70	77.8%

### Instruments

The respondents were asked to answer the Chinese version of Foreign Language Enjoyment Scale (FLES). Interpretations were provided for potential difficulties understanding the items since most of the participants were beginners of Chinese. The questionnaire started with a section to obtain participants' socio-demographic information such as age, gender, nationality and current location (whether in China or at home countries), followed by an additional question asking them to evaluate their own Chinese level on a 10-point scale of self-perceived achievement. All relevant inquiries were assigned to our participants in Chinese while explanatory or English instructions were provided if needed.

#### Foreign Language Enjoyment Scale

The Foreign Language Enjoyment Scale (FLES) in this study was adopted from Li et al. (2018) where the researchers transformed the original 21-item FLE scale in Dewaele and MacIntyre (2014) into a 14-item Chinese version. An 11-item scale remained after examination of the psychometric properties of the translated Chinese version of FLE. Responses were given on a standard 5-point Likert scale ranging from 1 (strongly disagree) to 5 (strongly agree), suggesting a possible score range from 11 to 55 points. A total score below 33, therefore, suggests little or no enjoyment, while a total score between 33 and 44 means the participants were going through middle level of enjoyment, and a total score above 44 indicates high level of enjoyment.

Li et al. (2018) provided significant construct validity [$\chi_{\left(41\right)}^{2}$ = 157.444; CFI = 0.980; TLI = 0.973; SRMR = 0.034; RMSEA = 0.041] after Confirmatory Factor Analysis (CFA) for the modified 11-item Chinese version of FLE. The general FLE scale and the subscales of FLE-Private, FLE-Teacher, and FLE-Atmosphere were all reported highly reliable (0.826, 0.792, 0.896, and 0.778, respectively). The split-half reliability was found to be 0.878, indicating high internal reliability of the scale. Strong convergent validity (CV) and discriminant validity (DV) were also detected. In this present study, the Cronbach's α for the FLES (Chinese version) was found to be 0.815.

#### Foreign Language Achievement

Participants' language achievement in this study was collected from two sources: self-perceived Chinese language achievement and scores from the actual final test at the end of the academic term. On the one hand, the respondents were asked to self-rate their proficiency in Chinese on the scale of 1–10 (1 means the lowest while 10 the highest). The same approach is also adopted in Young (1986), Liu (2018), Li (2019), and Liu and Yuan (2021). With a total score of 10, participants who achieve below 6, from 6 to 7, from 7 to 8 and above 8 were considered to be of low, lower-intermediate, intermediate and advanced level in their self-perceived Chinese language proficiency in this study, respectively. The final test, on the other hand, consisted of five individual subjects including Chinese listening and speaking, reading and writing, an HSK-model (standardized Chinese proficiency test) test, and two other optional academic subjects which were not related to Chinese language. Therefore, three test subjects (Chinese listening and speaking, reading and writing, an HSK-model test) were adopted as measurement of their actual Chinese language performance. With a total score of 300, participants who achieve below 180, from 180 to 240, from 240 to 270, and above 270 were considered to be of low, lower-intermediate, intermediate and advanced level in their actual Chinese language proficiency in this study, respectively.

#### Open Question

Following Dewaele and MacIntyre (2014), Li et al. (2018), and Zhang and Tsung (2021), this study also included an open question in a follow-up interview asking students to provide their descriptions of any episodes or events that they enjoyed in the online class. The question was asked in Chinese and participants were encouraged to answer in Chinese as well. However, English responses were kept optional lest some participants found it difficult to put their thoughts into exact words due to their possibly insufficient competence of the Chinese language.

### Research Questions

Starting from the perspectives of positive psychology, positive emotion and foreign language enjoyment with participants who were learning Chinese as a second language, the present study aims to investigate and answer the following questions:

(1) What are the levels of FLE for international CSL learners in an online learning context?
(2) How are CSL learners' FLE related to their foreign language achievement?
(3) Does participants' gender, nationality and presence in China have significant effect on their FLE?
(4) What are the possible sources of CSL learners' FLE?

### Procedures

Before administrating the questionnaire and relevant inquiries, we obtained consent from individual students and relevant third parties. Respondents were asked to finish the questionnaires *via* a Chinese online survey website *wenjuanxing* from June to July 2021. They were first approached in June by the first author *via* WeChat (a major social media application in China) or email for their consent to participate in this study since most of the respondents were not in China. Next, they were asked to fill out the questionnaires before their final test took place in early July. During the data collection process, altogether 101 preparatory language students were given the questionnaires while 98 of them finally managed to finish and complete submission on time. Eight of the finished questionnaires were dismissed due to inconsistent and inaccurate responses after review and examination of the data, leaving 90 valid questionnaires for further analysis. It needs to be mentioned that the term “Foreign Language” or “English Language” in some individual items of the FLE scale was rephrased into “Chinese Language” for better reference in this study. Finally, a follow-up interview was conducted by the first author to look for the possible sources of CSL learners' FLE in early September, 2021.

### Data Analysis

The quantitative data obtained in this study were all analyzed *via* SPSS 26. Descriptive and frequency statistics were first conducted to provide a general profile of CSL learners' FLE. Then, Pearson correlation analyses were run to examine the correlations among the following variables: general FLE and its subscales; FLE and learners' self-perceived and actual language achievements. The Tukey-Kramer multiple comparisons were employed to uncover the mean differences of FLE for learners of different FL proficiencies. A series of ANOVAs and *t*-test were conducted to examine the effect of gender, nationality and presence in China on participants' FLE. The qualitative data, obtained from follow-up interviews, were reviewed and sorted for possible sources of learners' FLE.

## Results

### Level of Foreign Language Enjoyment Among CSL Learners

Data obtained were run in descriptive analysis to provide the profile of the general scale and subscales of FLE among CSL learners. Tables 2, 3 report the results for parameters of mean score, minimum, maximum, mode, median, and standard deviation from the FLE.

**Table 2:** Descriptive statistics for FLE and its subscales (*N* = 90).

**Variable**	**Mean**	**Min**	**Max**	**Mode**	**Median**	**SD**	**Skewness**	**SE1**	**Kurtosis**	**SE2**
FLE	43.96	27	55	45	45	5.804	−0.651	0.254	0.207	0.503
FLE-P	20.07	10	25	19	20	3.172	−0.697	0.254	0.348	0.503
FLE-T	13.42	4	15	15	14	1.890	−1.784	0.254	5.539	0.503
FLE-A	10.28	3	15	12	11	2.656	−0.669	0.254	0.012	0.503

*FLE-P, FLE Private; FLE-T, FLE-Teacher; FLE-A, FLE-Atmosphere*.

**Table 3:** Descriptive statistics for each individual item of FLE (*N* = 90).

**Variable**	**Item 1**	**Item 2**	**Item 3**	**Item 4**	**Item 5**	**Item 6**	**Item 7**	**Item 8**	**Item 9**	**Item 10**	**Item 11**
Mean	3.82	4.14	4.42	3.44	3.79	4.42	4.42	4.46	4.54	3.26	3.23
Median	4	4	5	4	4	5	5	5	5	4	3
Mode	4	4	5	4	4	5	5	5	5	4	3
SD	0.931	0.801	0.779	0.901	1.044	0.779	0.703	0.752	0.656	1.232	1.039

The general FLE, as shown in Table 2, displayed a mean of 43.96, a median of 45, and a mode of 45. The subscales of FLE-Private, FLE-Teacher and FLE-Atmosphere in Table 3, respectively, had a mean of 20.07, 13.42, 10.28, a median of 20, 14, 11, and a mode of 19, 15, 12. The average parameters of the general FLE were 3.99 for mean, 4.36 for median and 4.36 for mode on a 5-point scale. All these figures suggested that our CSL learners experienced high levels of enjoyment in learning Chinese. FLE levels among CSL learners in this study proved to be higher than those in previous studies both in China and overseas (Dewaele and MacIntyre, 2014; Dewaele and Alfawzan, 2018; Li et al., 2018; Li, 2019). Participants also experienced significantly more FLE-Teacher (*mean* = 4.47) and FLE-Private (*mean* = 4.05) than FLE-Atmosphere (*mean* = 3.43). An observation of the correlation (see Table 4) between FLE and its three subscales has demonstrated that FLE-Teacher (*r* = 0.739, *p* < 0.05) and FLE-Private (*r* = 0.803, *p* < 0.05) were more positively correlated than FLE-Atmosphere (*r* = 0.707, *p* < 0.05) with FLE. This result suggests that, compared with language learning environment, students' self-perception of language achievement and teachers-related variables are stronger predictors of students' FLE.

**Table 4:** Correlations between FLE and its subscales (*N* = 90).

**Variable**	**FLE**	**FLE-P**	**FLE-T**	**FLE-A**
FLE	-			
FLE-P	0.803^**^	-		
FLE-T	0.739^**^	0.503^**^	-	
FLE-A	0.707^**^	0.314^**^	0.270^*^	-

** *Correlation is significant at the 0.01 level (2-tailed)*.

* *Correlation is significant at the 0.05 level (2-tailed)*.

*FLE-P, FLE Private; FLE-T, FLE-Teacher; FLE-A, FLE-Atmosphere*.

The standard deviations in Table 2 for the general FLE and subscales of FLE were 5.804, 3.172, 1.890, and 2.656, respectively, suggesting that there were great individual differences in students' experience of FL enjoyment. A further frequency analysis of FLE levels revealed that, of the 90 participants, 6 (7%), 37 (41%), and 47 (52%) students exhibited low (below 33 points), middle (between 33 and 44 points) and high (above 44 points) level of foreign language enjoyment, respectively. Some students even showed very high level of enjoyment in language learning while others felt little enjoyment.

### Correlations Between FLE and Language Achievement Among CSL Learners

It is of great pedagogical significance to look into the relationship of FLE and language achievement, and investigate whether positive emotions can help boost learners' better language performance. Prior studies (Dewaele and Alfawzan, 2018; Li, 2019; Li et al., 2019) have found significant positive correlations between FLE and language achievement in different learning contexts with various types of learners, suggesting that higher levels of enjoyment were linked to better language performance and vice versa. In this study, Pearson correlation analyses were conducted to investigate the correlations between CSL learners' foreign language enjoyment and their language achievement. The results, displayed in Table 5, informed us that our CSL learners' FLE was neither significantly correlated with their self-perceived language achievement (*r* = −0.007, *p* = 0.951) nor with their actual language achievement (*r* = 0.158, *p* = 0.137), suggesting that leaners' self-perceived and actual language achievement could be either high or low regardless of their FLE level. Similar results were found in Botes et al. (2020) and Chen et al. (2021). The former targeted at international undergraduate beginners learning Chinese in a Chinese university and reported no significant correlation between learners' FLE and their actual final language test performance, while the latter discovered no statistically significant interaction effect of self-perceived FL proficiency on FLE among multilingual learners of English, French and Spanish.

**Table 5:** Correlations among FLE, SPA, and AA (*N* = 90).

**Variable**	**FLE**	**SPA**	**AA**
FLE	-		
SPA	−0.007	-	
AA	0.158	0.112	-

*SPA, Self-perceived achievement; AA, actual achievement*.

Li (2019) found a significant positive correlation (*r* = 0.456, *p* < 0.001) between Chinese EFL learners' self-perceived achievement and actual language performance. She suggested that students who performed better in the actual test were inclined to be more positive about their own L2 ability and, alternatively, students who showed more positivity in their L2 proficiency tended to perform better in relevant L2 tests. Shao et al. (2013) and Yu et al. (2015) also uncovered similar findings (Li, 2019). The results (*r* = 0.112, *p* = 0.294) from this study, however, did not support the same findings.

**Figure 1:**
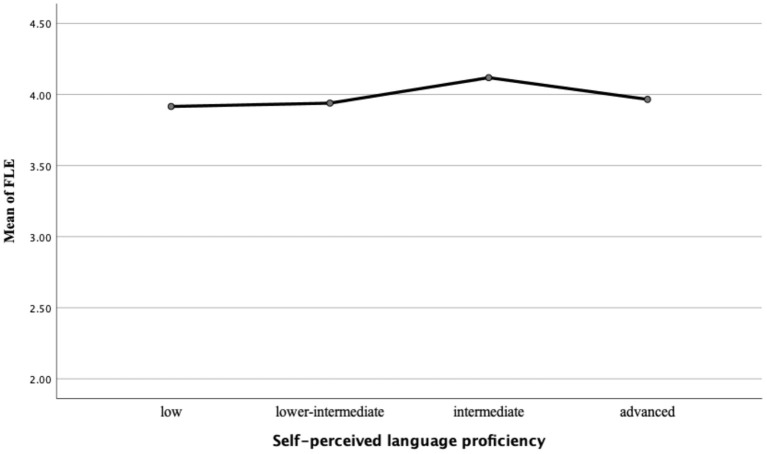
Means of FLE and levels of self-perceived language proficiency.

**Figure 2:**
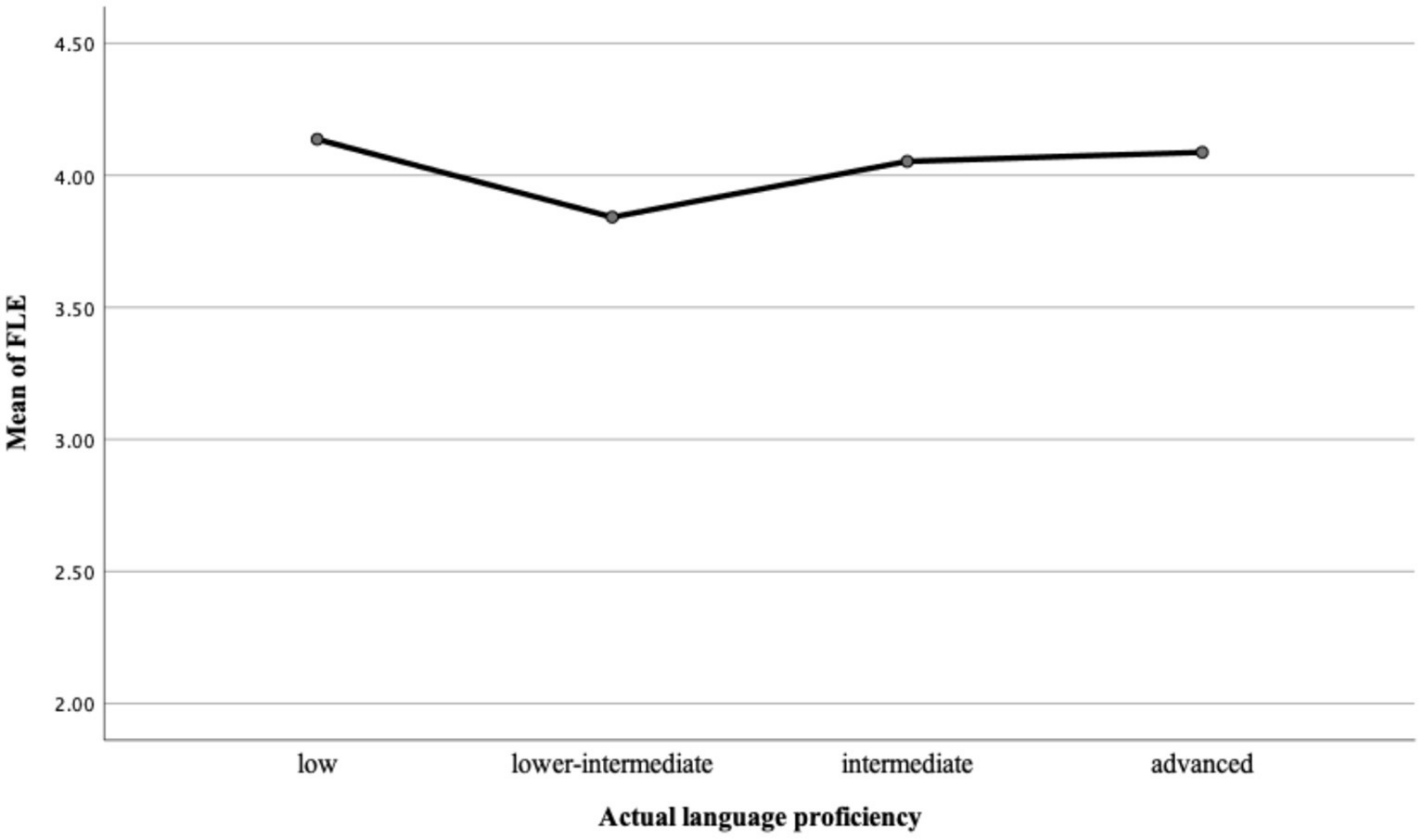
Means of FLE and levels of actual language proficiency.

Further analysis looking into the relationship between FLE and language achievement among learners of different language proficiency levels found a complex relationship. As far as learners' self-perceived achievement and actual language achievement are concerned, respectively, we divided our participants into four language proficiency groups: low (*N* = 26, 2), lower-intermediate (*N* = 21, 28), intermediate (*N* = 30, 42) and advanced (*N* = 13, 18) learners, according to the scores obtained from the self-perceived achievement report and the final tests. The Tukey-Kramer multiple comparisons indicate no significant mean differences (*p* > 0.05) of FLE for learners of different self-perceived and actual FL proficiency in this study as shown in Tables 6, 7. Figures 1, 2 show the means of FLE and the levels of CSL learners' self-perceived and actual language achievements.

**Table 6:** Multiple comparisons of self-perceived achievement and FLE (*N* = 90).

**Mean difference**	**Group mean**	**Low**	**Lower-intermediate**	**Intermediate**	**Advanced**
Low	3.92	-	−0.256	−2.223	−0538
Lower-intermediate	3.94		-	−1.967	−0.282
Intermediate	4.12			-	1.685
Advanced	3.97				-

**Table 7:** Multiple comparisons of actual achievement and FLE (*N* = 90).

**Mean difference**	**Group mean**	**Low**	**Lower-intermediate**	**Intermediate**	**Advanced**
Low	4.14	-	3.25	0.905	0.556
Lower-intermediate	3.84		-	−2.345	−2.694
Intermediate	4.05			-	−0.349
Advanced	4.09				-

As far as FLE and learners' self-perceived language proficiency are concerned, those who self-perceived themselves as intermediate experienced the highest level of FLE (*Mean* = 4.12, *SD* = 0.80), followed by those who self-perceived themselves as advanced (*Mean* = 3.97, *SD* = 1.07) and lower intermediate (*Mean* = 3.94, *SD* = 0.80). Participants who thought they were low achievers in learning Chinese felt the least enjoyment (*Mean* = 3.92, *SD* = 0.86). On the other hand, learners who actually scored low in their final tests and were grouped into low CSL achievers tended to feel the most enjoyment (*Mean* = 4.14, *SD* = 0.58) in this study. In contrast, low-intermediate learners were likely to experience the least enjoyment (*Mean* = 3.84.50, *SD* = 1.04). Little difference was spotted in FLE between advanced learners (*Mean* = 4.05, *SD* = 0.79) and intermediate learners (*Mean* = 4.09, *SD* = 0.77). The results from the two figures revealed that great disparities existed between FLE and CSL learners of different language proficiencies, indicating again that participants' self-perceived and actual language achievement could be either high or low regardless of their FLE level.

### The Role of Socio-Demographical Variables in CSL Learners' FLE

Socio-demographical factors are considered important variables that could influence learners' foreign language enjoyment, as well as foreign language anxiety, in the language learning process (Dewaele and MacIntyre, 2014, 2016; Dewaele and Dewaele, 2017; Dewaele and Alfawzan, 2018). Therefore, it is one of the purposes of this study to find out what role do socio-demographical variables, specifically gender, nationality and presence in China, play in CSL learners' FLE when they were attending virtual online classes.

#### Gender

A series of independent *t* samples tests showed that female CSL learners reported more FLE (*N* = 50, *Mean* = 4.05, *SD* = 0.87) than their male counterparts (*N* = 40, *Mean* = 3.94, *SD* = 0.87), which is consistent with previous studies that showed higher FLE among female learners compared with male learners (Dewaele and MacIntyre, 2014; Dewaele et al., 2016, 2017; Dewaele and Dewaele, 2017). In terms of the subscales of FLE, females scored higher in FLE-Private and FLE-Teacher (*Mean* = 4.16, 4.49, *SD* = 0.78, 0.76) but lower in FLE-Atmosphere (*Mean* = 3.41, *SD* = 1.14) than their male counterparts (*Mean* = 3.92, 4.45, 3.44, *SD* = 0.89, 0.63, 1.06). The results revealed no significant difference between gender and FLE, FLE-Teacher and FLE-Atmosphere (see Table 8). However, the results showed that there was significant difference between gender and FLE-Private [*t*_(*df*)_ = −2.451 (88), *p* < 0.05], suggesting that our female CSL learners were more concerned, than male CSL learners, about the positive feelings associated with their own achievement in language learning process.

**Table 8:** Independent *t-*test for FLE, FLE-P, FLE-T, and FLE-A between male and female CSL learners (*M* = 40, *F* = 50).

**Variable**	***F***	**Sig**.	***t* (df)**	**Sig. (2-tailed)**
FLE	0.547	0.461	−0.995 (88)	0.322
FLE-P	1.607	0.208	−2.451 (88)	0.016
FLE-T	0.334	0.565	−0.323 (88)	0.748
FLE-A	2.865	0.094	0.15 (88)	0.881

*FLE-P, FLE Private; FLE-T, FLE-Teacher; FLE-A, FLE-Atmosphere*.

#### Nationality

Our participants reported more than 20 different nationalities in this study, such as Japan, South Korea, Mongolia, Vietnam, Thailand, Russia, Armenia, Morocco, Turkmenistan, Kyrgyzstan, etc. In order to find out the difference of learners' FLE in relation to their nationality, we divided our participants into six general regional groups: West Asian (*N* = 7, 7.8%), Central Asian (*N* = 14, 15.6%), East Asian (*N* = 24, 26.7%), South Asian (*N* = 23, 25.6%), African (*N* = 10, 11.1%) and European (*N* = 12, 13.3%). A series of ANOVAs were conducted to find out the effect of nationality in this study. The results, however, showed that regional group did not appear to have a significant effect on CSL learners' FLE (*eta*^2^ = 0.048, *p* = 0.522).

#### Presence in China

It was also illuminating to see if CSL learners' physical location (whether they were in China or at home countries) could influence their FLE level since many of them were attending online classes at their own home countries where there was few, if any, occasions in which they could use the Chinese language. On the other hand, however, those who remained in China could use the language frequently in their daily lives despite still attending online classes.

Bearing this in mind, we analyzed the data and the results showed that those who were attending online classes at their own home countries reported significantly more FLE (*Mean* = 4.06, *SD* = 0.81) than those who were taking online courses in China (*Mean* = 3.76, *SD* = 0.95). Further analyses of the subscales of FLE among our learners revealed that students outside China scored higher in FLE-Private and FLE-Teacher (*Mean* = 4.18, 4.56, *SD* = 0.75, 0.60) but lower in FLE-Atmosphere (*Mean* = 3.38, *SD* = 1.14) than students who were in China (*Mean* = 3.61, 4.18, 3.58, *SD* = 1.16, 0.92, 0.95).

As can be seen in Table 9, the results of independent tests uncovered a significant difference between students' presence in China and their FLE, FLE-Private and FLE-Teacher [*t*_(*df*)_ = −2.451 (88), 3.411 (88), 2.402 (88), *p* < 0.05], suggesting that students who were attending online classes at their home countries, compared with those in China, were more concerned about self-perceptions of language achievement and that teacher-related variables were stronger predictors of their FLE. However, no significance was found between students' presence in China and FLE-Atmosphere [*t*_(*df*)_ = −0.9 (88), *p* = 0.37], which means that the language learning environment did not influence students' positive attitudes toward language learning.

**Table 9:** Independent *t-*test for FLE, FLE-P, FLE-T, and FLE-A between learners who were and were not in China (*N* = 90, *Yes* = 20, *No* = 70).

**Variable**	***F***	**Sig**.	***t* (df)**	**Sig. (2-tailed)**
FLE	0.928	0.338	2.333 (88)	0.022
FLE-P	1.065	0.305	3.411 (88)	0.001
FLE-T	2.775	0.099	2.402 (88)	0.018
FLE-A	0.85	0.359	−0.9 (88)	0.37

### Sources of Foreign Language Enjoyment

The analyses above have enabled us to investigate CSL learners' FLE from a quantitative perspective. However, qualitative data were also in need for the possible external or internal sources of CSL learners' FLE to get a deeper understanding of their language learning experience. To this end, 20 participants were invited for a follow-up interview. The interview included an open question asking students to describe their enjoyable moments in the class. A total of 1,182 English words were produced in the end with an average length of 59.1 words. After reviewing and sorting the answers, we found that they were consistent with the three dimensions of FLE in Li et al. (2018), i.e., FLE-Private, FLE-Teacher and FLE-Atmosphere. Table 10 summarizes the reported sources of FLE and frequencies of mentions, with reference to the classification standards in (Li et al., 2018).

**Table 10:** Sources of FLE in three dimensions mentioned by 20 participants.

**Sources**	**Content**	**Frequency**		**Percentage**
FLE-P	Sense of self-achievement in Chinese	7	28	56%
	Actual achievement in Chinese quiz or tests	4		
	Correct use of Chinese characters and grammar	2		
	Development of interest in learning Chinese	5		
	Using Chinese in peer interaction.	10		
FLE-T	Teacher encouragement, support and guidance in learning	4	13	26%
	Teacher non-traditional teaching instructions (e.g., use of games, role-play, movies, music, etc.).	9		
FLE-A	Group activities with positive engagement from teachers, peers and selves	8	9	18%
	Peer support.	1		

*FLE-P, FLE Private; FLE-T, FLE-Teacher; FLE-A, FLE-Atmosphere*.

#### Sources of FLE-Private

FLE-Private emphasizes the private pleasure coalescing around personal progress, excellent performance or interesting experiences in language learning (Li et al., 2018). Positive feelings generated from one's own ability of language learning was greatly valued as many participants in the follow-up interview ascribed their FLE to self-perceptions of achievement and actual achievements made in learning Chinese. Typical answers from student A, B and C are as follows:

Student A (female, 18): “I think I was the best in my Chinese classes, and I felt very happy that I got to learn what I want to.”

Student B (female, 20): “When my Chinese was understood by my classmates or teachers in the class, I would feel very happy. Also, I was more than excited when I knew that I passed the Chinese language proficiency test.”

Student C (female, 19): “I would feel the most enjoyment when I received good results from my Chinese tests, such as listening and writing exams.”

Some students also mentioned that the correct use of Chinese characters and grammar was conducive to helping them feel positively since the Chinese language is different from many other types of languages, especially Indo-European languages, in that it is composed of ideographic characters. Such ideographic characters often pose a great difficulty for students from other linguistic systems as they would struggle with writing the characters correctly. A typical answer from student D offers a view:

Student D (male, 19): “One of my best moments during Chinese classes was when the teacher was teaching a new word or new grammar that I was able to learn. Whenever I used that word or that new grammar correctly and people understood what I meant, I felt really happy!”

Learners' later development of interest in learning Chinese was also an important source of their FLE. Not a few participants in the interview mentioned that they found many interesting and meaningful things in Chinese, and they grew more interested in and became more positive toward learning Chinese. This was the view shared by student E:

Student E (female, 18): “Whenever I got the chance to do the reading assignments in class, I would feel the most enjoyable because I could learn so many things from them. I was always the first to finish the task and my teacher praised me for that, which made me grow fond of the reading tasks.”

Many occurrences of enjoyment reported by the participants see strong connection to the use of Chinese with peers when interacting with each other in class. They enjoyed communicating in Chinese and felt that it helped them know each other better, as express by student F:

Student F (female, 18): “I liked it when we got the chance to interact with each other in the class. It was nice to talk to them in the language we all knew. We talked about our hobbies and personalities. We liked to bring up our own topics, it felt like we were getting to know each other better.”

#### Sources of FLE-Teacher

Teacher is an irreplaceable role in educational activities. The second dimension of FLE, FLE-Teacher, highlights the enjoyable experiences related to foreign language teachers' supportive and encouraging attitude toward students, and their pedagogical practices (Li et al., 2018). As reported, CSL learners in this study thought highly of their teachers in terms of the encouragement, support and guidance they received in the learning process. A typical answer from student G is as follows:

Student G (male, 26): “I remember my teacher once told me not to feel sad about my (Chinese) test results and she told me that I was a good learner already. I really liked her saying that because it felt like I was valued and it meant a lot to me.”

Teachers' non-traditional pedagogical practice was another frequently mentioned source of FLE by participants in the follow-up interview. Students tended to enjoy the class more when the teacher employed various teaching aids, such as movies, videos or games, in conducting teaching activities. Student H and I expressed their thoughts as follows:

Student H (male, 18): “I enjoyed watching funny videos the teacher showed us during class, which kept the atmosphere light and fun.”

Student I (male, 22): “I liked the games that our teacher used in her teaching activities, because I liked learning through games. I really liked it.”

As Li et al. (2018) pointed out, FLE-Teacher overlaps, to some extent, with other two dimensions of FLE in that teachers play a central role in class and, therefore, they contribute either directly or indirectly to students' experience of FLE. Pishghadam et al. (2019) also redefined the role of teacher in the class from the perspective of emotioncy and regarded teacher as an “envolver” who exvolves or involves the learners for different purposes. Pishghadam et al. (2016a) listed three types of emotioncy: avolvement, exvolvement and involvement. The second teaching practice, exvolvement, was carried out through routine, book-related topics which can be dull and uninteresting by nature. Therefore, teachers would employ “various interactive, multimodal strategies to surpass single sensory learning and create a more engaging classroom setting compared with the traditional didactic lectures. Therefore, auditory, visual and kinesthetic emotioncies were developed by the employment of pictures, powerpoints, videos, etc. to promote (learners') cognitive processing” (Pishghadam et al., 2019, p. 48). In this study, nearly half of the participants inquired in the follow-up interview answered the questions with appreciation to their teacher's diverse instructional approaches and efforts in applying a variety of teaching tools or materials in the class.

#### Sources of FLE-Atmosphere

Sharing many similarities with prior studies on finding the sources of FLE (Dewaele and MacIntyre, 2014; Li et al., 2018; Zhang and Tsung, 2021), this study also confirmed that group activities involving positive engagement from teachers, peers and learner selves were a strong predictor of FLE. Participants reported that they felt a sense of belonging to the class owing to the group activities that brought teachers and students closer, and they believed that the group activities helped nourish a positive environment for students to know each other even in an online learning context. Typical answers from the participants are listed:

Student H (male, 18): “One of the moments I enjoyed the most was when the teacher divided us into groups and gave us topics to discuss, giving us the rare opportunity to get to know each other, since we are unable to meet each other in person due to the pandemic.”

Student J (female, 20): “I liked the group activities that our teachers and classmates all participated. We had a lot of fun. Everyone enjoyed themselves. I felt like we were a family. I am really lucky, happy and moved to have met them in my life.”

Student K (male, 18): “I remember one day when our teacher told us to bring a banana to class. The teacher divided us into groups to discuss how to peal bananas. Some students were invited to make demonstrations on ‘stage'. Their different and funny ways of pealing the banana made all of us laugh. I think the teacher was trying to teach us some life knowledge but I can't remember what that is.”

Peers' support was also mentioned, although only by one student, as a source of FLE. Student L appreciated the help from his classmates in helping him out in language learning and developed very positive attitudes toward his peers:

Student L (male, 25): “The first time I attended the online class, I felt like I was really bad at Chinese compared with my classmates because they could talk freely and knew exactly what the teacher was saying. But later I realized that they were all very nice and friendly. They would often help me when I had some problems in Chinese. I was really glad that I had support from them.”

A brief overview of the sources of FLE in three dimensions offers us the insight that learners' FLE is triggered by factors from a multitude of facets, many of which overlap with each other. As Li et al. (2018) put it, “the emotional state of foreign language learners is dynamic and complex, where a specific event could be triggered and influenced by interacting factors along different dimensions” (p. 193). It is therefore important for us to bear in mind that the three dimensions of FLE are inter-related and the separation of one from the other is not conducive to getting a full picture of learners' FLE.

## Discussion

The present study revealed that CSL participants experienced higher level of FLE in language learning than other FL, especially EFL, learners reported in previous studies. The results showed a mean of 3.99 for FLE among CSL learners in this study, which was higher than that of the international samples in Dewaele and MacIntyre (2014) and Dewaele and Alfawzan (2018) and the Chinese samples in Li et al. (2018) but was slightly lower than that of the CSL learners in Zhang and Tsung (2021). The possible explanation of lower FLE in this study, compared with that of Zhang and Tsung (2021), could be that most of our CSL learners were taking virtual online classes at their home countries and were not immersed in a Chinese cultural environment, and that our CSL learners were younger (average age = 20.14) than those (average age = 28.7) in Zhang and Tsung (2021), which was confirmed by findings in Dewaele and MacIntyre (2014) and Dewaele and Dewaele (2017) who found that FLE changed significantly between age groups with younger learners experiencing lower levels of FLE. Jiang and Dewaele (2019) found out that students' FLE was predicted more strongly by teacher-related variables. As can be seen in this study, the mean for each subscale of FLE, on a 5-point scale, is 4.47 for Private-Teacher, 4.05 for FLE-Private, and 3.43 for FLE-Atmosphere, partially reflecting that CSL learners' FLE in this study was also predicted more strongly by teacher-related variables (Jiang and Dewaele, 2019).

Dovetailing with the findings in Chen et al. (2021) who found no significant correlation between CSL learners' FLE and their actual language performance, the results of this study revealed that both CSL learners' self-perceived language achievement and their actual test performance were not significantly correlated with their FLE in an online learning context. This finding is inconsistent with previous studies that found a significant positive correlation between the two variables (Dewaele and Alfawzan, 2018; Li, 2019; Li et al., 2019). Comparing the findings between prior studies and this study, we found out that participants from the former studies were all advanced FL learners or were those who have studied the FL for a long time. However, in this study, the participants were almost beginners enrolled at an intensive Chinese-language training program designed to improve the proficiency of lower language level students. Li et al. (2019), after investigating FLE among Chinese EFL high school learners of different language proficiencies, found out that the predictive power of FLE on language performance was not significant among learners of low language proficiency. Therefore, the insignificant relationship between FLE and language achievement in this study, in contrast to previous ones, may be due to learners' lower linguistic proficiency, and, as Chen et al. (2021) states, FLE seems incapable of predicting any language achievement among language beginners (p. 50). Moreover, the participants from Dewaele and Alfawzan (2018) were mainly French, German, Spanish and English FL learners, while participants in this study were pursuing their studies of Chinese language. The linguistic difference between western and eastern languages could also be another possible factor leading to the disparities in research results. Further analyses of FLE among CSL learners at different language achievement levels also revealed that the relationship between the two variables was complex as participants' self-perceived and actual language achievement could be either high or low regardless of their FLE level. Another possible explanation for the absence of a link between FLE and language achievement in this study could be that the learners, mostly beginners, were highly motivated in language learning since they had to acquire good proficiency in Chinese during their language training process in one academic year and reach the language standard set for their future studies in Chinese universities. Such motivation and ambition may neutralize more transient emotions such as FLE. In other words, the relation between FLE and language achievement did not turn out to be significant because the learners enjoyed language learning but because they invested hugely in the mastering of the language, and not just in class but also outside.

Female participants in this study enjoyed their language learning experience to a higher degree than their male counterparts, as has been found in many prior studies (Dewaele and MacIntyre, 2014; Dewaele et al., 2016, 2017; Dewaele and Dewaele, 2017). Participants' gender was found to have significant effect only on FLE-Private in this study, suggesting that female CSL learners were more concerned about the positive feelings associated with their own achievement in language learning process. Although our participants reported a wide range of nationalities, their FLE were not found to be significantly influenced by the difference of their regional groups.

This study represents one of the first attempts to look at the effect of geographical location of international students on their FLE during the global COVID-19 pandemic that has denied their physical access to pursuing studies in a foreign country. The results showed that CSL learners who were attending online classes in their home countries scored higher in FLE, FLE-Private and FLE-Teacher but lower in FLE-Atmosphere than their classmates who were taking online courses in China. Such finding may be ascribed to the actual use of Chinese in our participants' lives. Speaking and having conversations in Chinese in real lives were not a current concern for most participants who were learning in their home countries as Chinese was not a medium for their daily communication and their use of Chinese language was more or less restricted to online classroom context in which teachers were their direct authentic providers of language feedback. Their temporary priority in studying was to complete the courses and tests without failure so that they could continue further studies in universities. Therefore, they had less direct communicative needs than those in China who were immersed in a Chinese-speaking environment and who faced putting the language in daily communication where they could meet troubles and difficulties, which may become a contributing factor to their lower level of FL enjoyment. Participants' presence in China was found to have a significant effect on their FLE, FLE-Private and FLE-Teacher, suggesting that students learning in online venues at home, compared with those in China, were more positive about self-achievement and developed more positive emotions toward their teachers. However, the effect of presence in China on FLE-Atmosphere was not as pronounced.

The sources of CSL learners' FLE in this study were analyzed from the three dimensions of FLE, i.e., FLE-Private, FLE-Teacher, and FLE-Atmosphere. Our finding confirmed Arnold (2011) and Dewaele and MacIntyre (2014) observation that students reported FL enjoyment when they perceived themselves as successful in language learning, as well as when they achieved good results from actual tests and exams. Teachers and peers also played vital roles in students' experience of FLE. Participants felt FL enjoyment when they received encouragement and support from teachers and peers, when they were engaged in group activities that fostered friendly atmosphere in which they could communicate with each other and develop interpersonal relationship. Teachers' non-traditional teaching practices were among the frequently mentioned sources of students' FLE in this study as students enjoyed the diverse approaches in class teaching to a great extent. Finally, the inter-related nature of different dimensions of FLE reminds us that a plethora of factors are intertwined in triggering students' experience of FLE and that we must adopt a panoramic perspective to view the sources of FLE.

## Conclusion and Implications

The present study examined positive emotion and foreign language enjoyment (FLE) among Chinese as a second language (CSL) learners attending virtual online classes in the emergency remote teaching (ERT) context amid the global COVID-19 pandemic. The findings revealed that our CSL learners showed high FL enjoyment when conducting language studies online and that female learners experienced more FLE than their male counterparts and gender was found to have a significant effect on one of the dimensions of FLE, FLE-Private. Despite no correlations announced between FLE and language achievement and between FLE and reginal groups, this study unveiled that participants' presence in China had significant effect on their FLE with those attending online classes in their home countries experiencing more FLE than those residing in China. The results of this study also highlighted that teacher-related variables and learner self-perceptions of achievement strongly predicted CSL learners' FLE.

Apart from the results obtained, this study also has important limitations and provides ample opportunity for future research. The first and foremost limitation is the relatively small number of participants. This study involved learners in a preparatory intensive language training program in some local university in Shanghai, China. The pandemic has greatly reduced the number of students participating this program for this year due to the fact that overseas students could not come to China to study and many of them were unwilling to take online classes. Many have decided to take a gap year or drop out. As a result, the author could only approach a limited sample of learners. Secondly, although a high level of FLE among CSL learners was found in an online learning context in this study, the lack of pre-pandemic data of their FLE level in offline face-to-face classes prevented us from conducting further analysis and comparisons. We know that our participants experienced high level of FLE in an online learning context but we could not ascertain if it was higher than that of an offline face-to-face learning context. Therefore, we cannot conclude with affirmation that online classes have helped boost learners' FLE or reduced their FLE. On top of that, we only focused on a few socio-demographical variables, such as gender and nationality, in this study, and we also failed to include participants from the Pacific and American regions.

Nevertheless, this study still yields a number of theoretical and practical implications concerning the teaching and learning of Chinese as a second or foreign language both in China and in a global context. Theoretically, we focused on positive emotion that has long been ignored and downplayed due to the research preference on negative emotions, and involved Chinese as a second language (CSL) learners as research subjects since few emotion studies in SLA have targeted this type of learners. We looked into foreign language enjoyment among CSL learner who were learning the language in an emergency remote teaching (ERT) context during the global COVID-19 pandemic period. Their high level of foreign language enjoyment demonstrated that the CSL learners developed great amount of positive emotions in their language learning process and that they were highly positive toward learning the Chinese language, highlighting the important role of positive emotions in positive psychology played in language teaching and learning. Practically, foreign language teachers, CSL teachers in particular, should be well aware of their role played in helping boost students' positive emotions, and make the most of students' positive emotion to facilitate their language learning. CSL teachers should bear in mind that although positive emotion may not necessarily boost learners', beginners' in particular, language performance in the short term, it can stimulate their learning interest and help them develop a sense of belonging to the language or culture, which will in turn expedite language learning in the long run.

To conclude, the present study has highlighted that FLE will benefit beginning CSL learners in the long run and that teacher-related variables and learner self-perceptions of accomplishment are great contributors of FLE. To enhance better understanding of FLE among CSL learners, future researches should look deeper into the relationship between FLE and other socio-demographical variables such as learner motivation, and involve CSL learners at higher language levels. Recommendations are also given to studies investigating foreign language anxiety (FLA) and its relationship with FLE among CSL learners, particularly in an offline “in-person” teaching context.

## Data Availability Statement

The original contributions presented in the study are included in the article/supplementary material, further inquiries can be directed to the corresponding author.

## Author Contributions

All authors listed have made a substantial, direct, and intellectual contribution to the work and approved it for publication.

## Funding

The paper was supported by the research project University English Teachers Thinking and Practice Routes in the Integration of Ideological Education into Course Teaching in the Context of the New Outlines (Grant Number: C2022405), sponsored by Shanghai Municipal Educational Science Planning Project; the research project Municipal Key Courses of the Universities in Shanghai–English Curriculum and Pedagogy, sponsored by Shanghai Municipal Education Commission; and the project Exploring the Route and Measures of the Integration of Critical Thinking into English Language Teaching, sponsored by the Shanghai Centre for Research in English Language Education.

## Conflict of Interest

The authors declare that the research was conducted in the absence of any commercial or financial relationships that could be construed as a potential conflict of interest.

## Publisher's Note

All claims expressed in this article are solely those of the authors and do not necessarily represent those of their affiliated organizations, or those of the publisher, the editors and the reviewers. Any product that may be evaluated in this article, or claim that may be made by its manufacturer, is not guaranteed or endorsed by the publisher.

## Appendix

To what extent do you agree with the following statements?

Strongly Disagree/Disagree/Undecided/Agree/Strongly Agree.

**Table A1:** The Chinese version of the modified 11-item Foreign Language Enjoyment Scale (FLES), adopted from (Li et al., 2018).

***N***	**Content (Chinese)**	**(English)**
1)	我不厌倦汉语学习。	I don't get bored.
2)	我享受汉语学习。	I enjoy it.
3)	学汉语的过程中, 我学了很多有趣的事情。	I've learnt interesting things.
4)	在班里, 我为自己的汉语成绩感到自豪。	In class, I feel proud of my accomplishments.
5)	周围汉语学习的氛围很好。	It's a positive environment.
6)	学汉语很有趣。	I's fun.
7)	老师总是鼓励我们。	The teacher is encouraging.
8)	老师很友善。	The teacher is friendly.
9)	老师总是支持我们。	The teacher is supportive.
10)	我身边有很好的汉语学习氛围。	There is a good atmosphere.
11)	我们有紧密的学习小组。	We form a tight group.

*FLE-Private: item 1, 2, 3, 4, and 6*.

*FLE-Teacher: item 7, 8, and 9*.

*FLE-Atmosphere: item 5, 10, and 11*.
